# Thinking Time, Shifting Goalposts and Ticking Time Bombs: Experiences of Waiting on the Gender Identity Development Service Waiting List

**DOI:** 10.3390/ijerph192113883

**Published:** 2022-10-25

**Authors:** Kathy McKay, Eilis Kennedy, Talen Wright, Bridget Young

**Affiliations:** 1 Public Health, Policy and Systems, Institute of Population Health, University of Liverpool, Liverpool L69 3BX, UK; 2 Tavistock Research Unit, Tavistock and Portman NHS Foundation Trust, London NW3 5BA, UK; 3 Department of Clinical, Educational and Health Psychology, University College London, London WC1E 6BT, UK; 4 Division of Psychiatry, University College London, London WC1E 6BT, UK

**Keywords:** waiting list, waiting times, gender identity, mental health, wellbeing

## Abstract

LOGiC-Q is a prospective longitudinal qualitative study that explores the experiences of children and young people, and their families, who have been referred to the Gender Identity Development Service (GIDS) in the UK. This paper describes the experiences of children and young people and their parents while they are on the waiting list to be seen. Semi-structured interviews were undertaken with 39 families who had been referred to GIDS and were waiting for their first appointment with the service. Both parent and child/young person were interviewed. Analysis of the anonymised interview transcripts was informed by both narrative and thematic approaches, and three predominant narratives around waiting were identified: 1. Positive experiences attached to waiting; 2. Feelings of distress and stuckness; 3. Suggestions for support while waiting. Findings from this study indicate variations in how waiting is experienced depending on the age of the child, and how distressed their body makes them feel. Young people and their parents offered suggestions for how the service could support families on the waiting list. These suggestions related primarily to ways of checking in and providing reassurance that they were at least still on the list as well as ideas about how to make the wait less distressing, rather than necessarily making the wait shorter, which was more spoken about in terms of an ideal rather than a realistic option.

## 1. Introduction

The experience of waiting is arguably universal, something that encompasses commonplace activities (waiting for the phone to ring) to more unusual activities (waiting for an organ transplant). As waiting is universal, so too are the positive and negative emotions attached to the experience–anticipation and anxiety, relief and frustration, excitement and fear. Not every emotion will be felt by a person who is waiting, and some emotions will be more intense and felt for longer than others. These feelings depend on factors including the age of the person waiting, what they are waiting for, where they have to wait, and the length of time they have to wait and what happens to them during this time. Waiting may be a very normalised experience, but it can still be a painful experience that can have lingering impacts on an individual’s wellbeing [1].

Waiting is commonly experienced by people within the healthcare system, by patients as well as their family. Studies looking at the impact of waiting on people who needed coronary artery bypass surgery [2] and kidney transplants [3,4] highlighted the anxiety, powerlessness, and frustration felt. Participants in one study involving patients waiting for cardiac surgery “felt that their lives were on hold, and that they could not make plans for the future as a result” [2] (p. 1246). Those waiting for a kidney transplant had similar feelings, accompanied by fears not only about whether they would match with a kidney before their illness progressed too far but if the transplant itself would be successful [3]. These fears also affect the families of those waiting, especially when the person waiting is a child [5] (p. 344).

Waiting therefore becomes more than simply being on a list and waiting one’s turn. People waiting for treatments for chronic and terminal illnesses often felt they had to be a ‘good’ patient so as to not hinder their access to treatment [6] (p. 1062). While waiting for treatment following a cancer diagnosis, Mulcahy and colleagues [6] found that people could swing between feelings of powerlessness, especially when waiting times began to feel endless, and more powerful feelings, where the patient might be proactive in seeking care either outside the public or conventional health system altogether.

Indeed, Ferrie and Wiseman [7] found a ‘good’ patient was presumed to be able to endure any wait for their treatment, but their wait could also be shaped by privilege, affected by the patient’s resources and socio-economic status. Those who can afford to access private treatment will not generally have to wait as long as someone dependent on the public healthcare system. Patients receiving private care often have agency over how long they wait and in which system, which makes their experience arguably more hopeful than if they were just relying on being in a queue without any choice in the latter. In contrast “waiting without choice or knowledge was distressing for participants. As this process seemed to increasingly stretch and protract time, potential for hope simultaneously began to diminish” [7] (p. 10). Here, the ways in which healthcare professionals communicated to people about their wait (primarily how long it would be and what it would entail) could help to preserve hope even if the length of time a person was on a waiting list could not be shortened.

However, people were more vulnerable to feelings of hopelessness and despair if there was a lack of clarity or transparency about the waiting list and what it entailed; or when there were multiple waits within a system (for example, waiting to see a physician for a referral and then having to wait to see the next physician) [8]. While potentially harmful to a person’s wellbeing, people worked to find ways of coping with their anxiety about when they would receive the care they needed: “Participants described waiting as something uncertain, imposed on them by systems and individuals in ways that were out of their control, and yet they also understood waiting to be something to manage” [8] (p. 5).

Better understanding of how the stresses of waiting can be managed can help services support patients, and their families, even before an intervention begins. Sweeny and Cavanaugh argue that strategies based in “uncertainty navigation” are effective in dealing with the anxieties of waiting [9] (p. 147). These strategies are grounded in: “(1) consequence mitigation; (2) reappraisal; and (3) emotion regulation” [9] (p. 148). However, Sweeny and Cavanagh [9] noted that the effectiveness of these strategies is not long-term and some may have negative consequences. In this way, the longer a person has to wait the more likely it is that their wellbeing will be significantly negatively impacted.

Long waiting lists for children’s gender identity services, where children and young people can access gender-related care from qualified specialists from both physical and mental health fields, have become both normalised and subject to criticism in many countries. Young people in a Canadian study spoke about the frustration they felt in how difficult accessing care could be, describing it as a “long, winding, and complicated path” that involved family and healthcare professionals, even when the support they eventually received was positive [10]. Similarly, research examining patients’ and parents’ experiences of the Royal Children’s Hospital Gender Service in Australia showed significant unhappiness with the lengthy waiting time of >12 months, but also significant satisfaction with the service once they were able to access support there [11].

Waiting and its impact on wellbeing is something that is increasingly being considered in relation to lengthy waits for children and young people wishing to access children’s gender identity development services in the UK. Long waiting times are also acknowledged to be an impediment to accessing timely care in in adult services [12], and can also lead to parents of children and young people feeling isolated while on the waiting list for young people’s services [13]. This paper from the NIHR funded LOGiC-Q study (Longitudinal Outcomes of Gender Identity in Children-Qualitative study) describes the experiences of children and young people and their parents while they are on the waiting list to be seen at a national specialist gender service. We describe how waiting for treatment affects the lives of children and their families. We also report how families work to mitigate the challenges associated with waiting and the suggestions they provide regarding the types of support that might be helpful to families while waiting.

## 2. Materials and Methods

### 2.1. Study Design and Ethics

LOGiC-Q is a prospective longitudinal qualitative study that explores the experiences of children and young people, and their families, who have been referred to the Gender Identity Development Service (GIDS) part of the National Health Service (NHS) in the UK. LOGiC-Q is part of a larger mixed-methods programme of research and is a companion qualitative study to a longitudinal cohort study, the ‘LOGiC’ study. At the time of study recruitment, GIDS was the only centre that treated children and young people in England and Wales [13]. Protocols for the LOGiC study and LOGIC-Q are available via open access [14,15,16].

Ethical approval was granted by the UK Health Research Authority and London–Hampstead Research Ethics Committee as application 19/LO/0857. Given the sensitive nature of the study and the potential vulnerability of the families involved, the qualitative team has also endeavored to keep the entire methodological process in line with the recommendations set out by Vincent [17] to ensure the safety and agency of all the participants and everyone else involved in the study.

### 2.2. Participant Recruitment and Consent

Participants for LOGiC-Q were recruited from the main longitudinal cohort study, the LOGIC study. Families whose child had been referred to the GIDS waiting list when they were aged 3–14 years, were contacted by an administrator from the service who sought their consent to be contacted by a researcher in the LOGIC study team about participating in the study. Families who agreed to participate were then asked for consent for their contact details to be passed to the LOGiC-Q researcher (KM). Contact details of 97 families were passed on to KM, who contacted 78 families who met the purposive sampling criteria. These criteria aimed to ensure a diverse sample in terms of child age, gender, transition stages-fully socially transitioned (young person living as their identified gender in all aspects of their life), partially socially transitioned (young person living as their identified gender in some aspects of their life but not all), and no social transition (young person still living as their birth-assigned gender)-Autism diagnosis, ethnicity, geographic location, and socio-economic status. Of these 78 families, 39 (50%) were interviewed. Of the remaining 39 families most (N = 23) did not respond to KM’s contact. Dates could not be set with nine families and one parent declined due to timing. After COVID-19 lockdown restrictions were put in place in the UK, six families did not want to do an interview over zoom/phone and so were unable to be included. Recruitment ended at 39 families as information power was reached [18].

Before interviews, KM went through the age-appropriate information sheets with families and answered their questions. She stressed that participating was entirely voluntary and that both the parent and child/young person could choose to not answer a question or stop the interview at any time without worry. KM took particular care explaining these details with the younger children, showing them how a recording was made and how it could be deleted, and ensuring that they felt comfortable in refusing to answer a question or asking for a break.

### 2.3. Participants and Lived Experience Inclusion

We wanted to ensure that the LOGiC-Q participants included varying experiences of gender identity and transition, as well as belonging to an ethnic minority identity and whether the young person had been diagnosed with Autism. The families lived in both urban and rural areas in England and Wales, and we also worked to ensure they were from a range of socio-economic areas. We used the English Indices of Deprivation 2019 to judge this; we accessed neighbourhood level data using postcodes. Areas are ranked from 1–10 in terms of deprivation with 1 being the most deprived 10% and 10 being the least deprived. The participants’ pseudonyms and some basic demographic data about them is included in Table 1.

Thirty-nine families equated to 78 participants (primarily a mother and her child) with occasions where another parent or sibling would join the conversation. We want to highlight that we have included the pronouns used by the young people at the time of the interview, but not their birth registered sex. This was decided after KM talked with the older teenagers participating in the study after their interview, TW (lived experience co-investigator), as well as the LOGIC study PPI group. It was clear from these discussions that birth-registered gender was not something the young people wanted to be identified by and so we have respected their wishes.

### 2.4. Interviews

Audio-recorded semi-structured interviews were undertaken between July 2019 and June 2020, a time that encompassed the first COVID-19 lockdown in the UK. Prior to the March 2020 lockdown, 27 interviews were in person; afterwards, 11 interviews were conducted over Zoom and one by phone. In line with our focus on ensuing all participants felt safe while talking to KM, we offered families the choice of whether to be interviewed separately or together. Indeed, some children and young people asked whether they could speak together with their parent before KM asked. In total, parents and children/young people in 28 families chose to be interviewed together, while those in 11 families chose to be interviewed separately. Interviews lasted between 11 to 103 min (average 46 min); times differed between shorter individual interviews with younger children and longer interviews with parents and teenagers.

Interviews were conversational and informed by the topic guide developed by the authors. KM started by asking parents to describe their children and for the child/young person to describe themselves. Questions followed the parents’ and child/young person’s journey from when the young person first identified as gender non-conforming (in whatever form this took), to when they first told someone in their family, to accessing the GIDS waiting list, and what they had experienced in terms of support in the interim. When interviews were undertaken separately, KM did not disclose anything that the first person had said to the second person being interviewed. When interviews were done together, especially with teenagers, there was often discussion between the two participants comparing differences in experiences, and differences in interactions with services and schools, and hopes and expectations for the future.

Given the sensitive nature of some of the topics of conversation, and the young age of some participants, KM took numerous steps to help participants feel comfortable during interviews and support them in voicing their perspectives. The time and location of the interviews were always chosen by the family, and could be rescheduled as needed. KM often demonstrated the recording device with younger participants to show them how their voices sounded. Stuffed toys were included in the interviews with younger children, as well as letting people tell their stories in their own time and in their own way. When interviews were done over Zoom, some younger children drew pictures during interviews to help them feel more comfortable talking virtually. Appearances from KM’s cat, as well as the pets of the participating families, helped make the interviews feel more informal and relaxed. In addition, if the parent or young person reported distress, KM sought permission regarding potentially passing on their details to the service to arrange additional support. Aware that the conversations with families covered sensitive and personal experiences, and especially if the narratives had been distressing, KM took great care to ensure interviews ended on a positive note. In situations where distress was identified, KM would follow up with families with regard to support being put in place. KM made sure to be guided by the children and young people particularly, as well as the parents, working to not assume, pathologise, or dichotomise experiences [19].

### 2.5. Analysis, and Reflexive and Inclusive Practices

After each interview, KM wrote reflective field notes describing her perceptions of the interviews, important points that had been brought up, and (dis)connects between different families’ narratives as the interviews progressed [20,21]. KM also debriefed with EK after interviews that required referral for additional support. The audio-recordings were transcribed verbatim, then anonymised. KM listened to all the audio-recordings alongside the transcripts to check their accuracy. Analysis of the anonymised interview transcripts was informed by both narrative and thematic approaches [22,23,24]. In line with the guidance around reflexive thematic analysis set out by Braun and Clarke [22], KM read and re-read the transcripts, taking notes, and organically coding across and within the narratives. The field notes also helped to provide context to the narratives. This reflective approach also involved actively collaborating with various groups. KM regularly met with the co-authors to go through different transcripts and discuss potential themes. KM also regularly met with the LOGIC PPI group, made up of parent/carer, child and young person participants from the entire LOGIC cohort study, to discuss study design and management and preliminary findings, including those examined in the current paper. Feedback was also provided to clinicians within the service with aim of informing service provision by taking into account perspectives from families. Using these reflective and collaborative practices helped us to ground the analysis in the lived experiences of the participating parents, children, and young people. We use the word ‘parent’ in this paper to mean any adult in a caring role to help maintain the anonymity of participants. All parents, children, and young people have been given a pseudonym.

## 3. Results

While LOGiC-Q involves interviewing families longitudinally over a number of time-points, this article reports on the experiences of waiting that were described during the first interviews. At this time, only one parent had attended an appointment with GIDS and it had been their first appointment. Other families had been waiting between 4–24 months without an appointment.

Three predominant narratives around waiting were identified:

1. Positive experiences attached to waiting;
2. Feelings of distress and stuckness
  - Uncertainty and fear attached to puberty
  - Fear of missing healthcare milestones and aging out
  - Cannot see a future while they’re stuck waiting; and,
3. Suggestions for support while waiting.

### 3.1. Positive Experiences Attached to Waiting

Some families identified positive aspects to being on the waiting list. Parents who felt they were close to the end of their wait, thought waiting had “given us all breathing space” (Maryka, parent), and suggested that the time helped in reaching certainty about their child’s gender identity: “this is why process is so long isn’t it, to make sure… you know that decision is never going to change” (Marianne, parent). One parent thought that feeling positive about waiting was not a common experience: “Probably the first people in history who are pleased for a waiting list! [laughs]” (Aimee, parent).

Positive experiences were most common among families with younger children, where there was more time for the child to receive care from a gender identity service before puberty. Waiting times were well known among the participants and was one of the reasons many parents of younger children had not waited long before trying to access the waiting list:

And we were already quite well informed of waiting times… which is why we’ve gone for it this early. So when the time comes, it’ll be just about the time that [Alana] will be sort of thinking about the next stage. So you know we’ve kind of timed it as best we can for that.

(Bonnie, parent)

We’re not scratching at the door… we just did it because we heard that it’s easier to be on to be in the system… So that if he should need medical intervention then there’s a long history of them having known us.

(Pippa, parent)

Indeed, there was a palpable relief among some parents that they had been able to act as early as they had, because the waiting was stressful even at a young age:

I’m so pleased that he could feel he could tell us at such an early age, for us to be there so early on Tavistock… Because some days I’ll just stand there and panic and think you know what if weren’t so early? What if we weren’t in this position to be on Tavistock so early that we couldn’t be there before we need the blockers. There before he needs the testosterone…

(Ingrid, parent)

Younger children (5–10 years of age) did not express worry about the waiting list, and it was unclear how much they understood of its significance. Some of their parents though expressed future-facing anxiety about whether their child would be able to continue ‘passing’ as the gender they identified with, once puberty started and had their body physically changed as a result. One parent worried that: “I just don’t want anything missed… if she’s going to be a girl, she stands a chance [but] if we start getting… any Adam’s apple or anything like that… she won’t have a chance of looking normal… whereas now at least she’d pass completely as normal” (Clara, parent). Another parent, April, was concerned about making decisions for their child who at the time of the interview, “whilst he’s seven, probably developmentally and emotionally he’s more like a four year old just because of his needs” (April, parent). April spoke about how she and her partner tried not to worry about the future, but also of their reluctance to make important decisions on their child’s behalf:

So we wouldn’t want to make a decision that affected his future… that’s why we probably try not to worry about it, but I know we might get to a point one day where we’re like oh… [child] talks about having kids when he’s older and… I really don’t know whether I’d want to make a decision. Because obviously if we said oh yes he wants to be a girl and go through all this… And then he couldn’t have kids and one day him going ‘Why did you do that to me? Why?’ [laughs] … because it’s a long time in the future and then anything could happen. But I think that would be our worry. Because I think [husband] and I aren’t keen to make those kind of decisions.

(April, parent)

In this way, the waiting time gave April space before having to make any of these decisions.

In comparison, older children and teenagers were very aware of the time they had been on the waiting list and were often concerned about it. At times this meant that the parent and child did not see the wait in the same way. Ines was more positive about Dylan still being on the waiting list than he was as he had started puberty, and they discussed this disconnect together. Please note that Dylan preferred he/him pronouns which Ines still struggled with throughout the interview:

[Ines, parent]: to me she’s still a child and I don’t think they should have that option until they’re eighteen at least… I know she probably won’t ever change her mind … but it’s… at the same time I’d rather her develop first… rather than stop it! [Ines laughs because Dylan has raised eyebrows at this]

[Ines]: She doesn’t agree with me at all… she’d rather stop that in its tracks right now, isn’t it?

[Dylan, young person, 14]: Yeah.

[Ines]: Before it goes any further like …

[Dylan]: Yeah. Well I… just try not think about it too much… Distract… yeah, just don’t think.

[Ines]: She doesn’t like to dwell on things. We’re not allowed to talk about it, it’s a dirty word!

[Dylan]: No.

Parents and teenagers sometimes had different perceptions of how the waiting time had passed. Rose and Davie explored how their experience of waiting had changed over the nearly two years Davie had been on the waiting list. They had normalised the waiting so that the experience would not be too emotionally devastating:

[Rose, parent]: For me it’s really gone fast, but I think [Davie] is terribly frustrated.

[Davie, young person, 12]: But it’s like every day… like in the car on the way home I think about what if I’m there and the letter’s there when I’ve got in and whatever. But like it’s kind of just got like a normal thing now, it’s not… I can… I’m not mad about it. It’s just frustrating having to wait, but I understand why I have to wait, but it’s just frustrating.

[Rose]: At first you didn’t did you?… It was tantrums and tears, why can’t they have it now, why can’t they have it now!

[Davie]: [laughs]

### 3.2. Feelings of Distress and Stuckness

Feelings of distress and stuckness were most prominent in the narratives of young people close to or already experiencing puberty and who had not yet had their first GIDS appointment. They struggled to see a future beyond being able to access the support they wanted at GIDS and this caused significant distress:

“Oh God, it’s awful! Because I… I’m a very impatient person, so waiting it just kills me… Like I… I knew it would take a long time, but it’s going to take a very long time, but waiting is just… you know and I know that you’ll get there one day, but you never know when and so you’re just stuck there waiting. Like you can’t do anything until you’re finally there”.

(Lucian, young person, 11)

Some teenagers, spoke about the length of time they had been waiting, and the stuckness they felt, almost jokingly perhaps, as a way of coping with their feelings:

Remember when I first figured out I was trans and it was just before like… I think I said it’s within a few months or something and, um, I was telling my friend [name] and I was like oh maybe when I come back from the six weeks you’ll see me with a beard and I’ll be on T. And now I look back and I’m like… [Frankie] what were you thinking? [laughs]

(Frankie, young person, 15)

Being able to make light of their past expectations did not necessarily limit the hurt these young people felt at having waited for so long for a GIDS appointment. Indeed, the parents of a younger child were worried about the length of the wait, especially as they were also waiting for other mental health assessments. Tom and Clara were trying to positively frame the time waiting for support for their child on the advice of an older transgender woman they had met at a family group:

“[Tom, parent]: She said ‘yeah it feels like forever and you feel like it’s never going to get anywhere, but… It does happen… [laughs]

[Clara, parent]: Everything for [child] poor lamb is… is such a long way …”

*a*. *Uncertainty and fear attached to puberty*

Young people and their families had little way of judging how soon they might receive an appointment other than comparing their wait times to others. This was not a fool-proof method as participants had little information about how many referrals were made to the gender service at any given time. The constant comparison created another type of stress for young people in trying to work out a timeline to not feel as though the wait was endless, and to buoy their own wellbeing, but to also not create any false hope. Young people were also balancing wanting to contact the service for information about how much longer they might have to wait and not wanting to be seen as a bother:

It’s hard to kind of keep your dysphoria at bay when… you’ll be here in a few months and that’s guaranteed because obviously the waiting lists have like skyrocketed in the past few years… which is really frustrating! [laughs] … I’m just chilling at the moment with all of it, um, but there have been points where I’ve kind of got a bit distressed about the whole waiting list thing. I’ve had to call up and be like how much longer will I have to wait? [laughs]… So I haven’t done that a lot because obviously I don’t want to be bothering people when I’m not supposed to.

(Bodhi, young person, 15)

Some parents felt the only way to get through the wait was to regularly contact the service to see if they could gather any further information about how long their wait might last. While parents were frustrated they understood that the waiting times were so long because the service was caring for an increasing number of young people:

The need has overtaken the supply (umm) massively… it’s a big tidal wave… But I have made a mental note to ring them again next week and check in and see where they’re at because (umm) you know if I keep ringing perhaps I’ll just get sick of me ringing! [laughs] So I will try again next week.

(Caroline, parent)

The fear attached to puberty for many of the teenagers was that while they might be stuck waiting for their first appointment, their body was developing in ways that made them feel anxious and ‘other’. Rose and Davie spoke together about this:

But the thing that I’m more bothered about is all this time while we’re waiting, we understand why we have to wait… I mean… but lots of reasons why we have to wait, but all this time he’s developing”.

(Rose, parent)

It’s really annoying because… like all these boys at school have… because I’m in year eight now. So like second year of secondary school. And all these boys’ voices are dropping and they’re growing like jawlines and… and getting muscles and… little beards… and I’m kind of stuck there and I… I know my voice is really like feminine, well… well I think it is.

(Davie, young person, 12)

Fear of puberty was intense with both young people and their parents describing it as ‘a time bomb’. One parent worried the beginning of puberty would spell the end of her child’s happiness, “because she’s happy at the moment, it’s not a problem. But I’m worried about the development, because that’s a time bomb, there’s nothing we can do to stop the development” (Brianna, parent). Amara spoke about how waiting without being seen by the service meant that she had no control over the changes puberty would bring to her body, which she worried would make her look and sound less feminine than she wanted. Amara had a very clear image of how she wanted to look as an adult in terms of height and how her voice would sound. She talked of the ‘ticking time bomb’, and was anxious that her body would change, she would grow irreversibly and never be able to look how she wanted to look:

It’s like a ticking time bomb… so it’s kind of like everyone is taller than me, but I’ve had a fear of just like all of a sudden just growing… And like not being like short anymore. Because like I want to be five foot four… I don’t want to be anything over five foot five.

(Amara, young person, 12)

Amara added, “if you’re already eighteen, you’re fully grown and you won’t like shrink”.

One source of hope in the midst of this distress was that young people would be able to access puberty blockers once they were seen by the service and assessed as being eligible for them. While many parents still had questions about their child taking puberty blockers, they saw the possibility of being able to pause puberty as beneficial because they gave a young person time to “find out” what they want:

Puberty blockers would be absolutely brilliant. As far as I know, I haven’t done much research on them. I don’t know if they block other things. They might block height development for example which is… would be something to think about. But I’ll take anything I think at the moment to buy her time to find out what she wants… And we can’t get the puberty blockers without GIDS because nobody will prescribe them.

(Brianna, parent)

Puberty blockers were “a priority” for Marianne and Iris because Iris’s mental health had suffered as puberty had started. Marianne believed that puberty blockers would give Iris space where:

[Marianne]: You’re content in waiting. And you’ve got that peace of mind and for me the mental health… the… like when [Iris]’s having a really rough time the damage it does to the mental health is absolutely… it’s just…

[Iris, 11]: Horrific.

[Marianne]: It’s absolutely heart-breaking and I phoned GPs saying look is there anybody that I can get her in with counselling, anybody that can help… they’re like… no, we don’t know of anything. (sigh) … It’s just been a battle… Just keeping tapping away at it, well I’m just trying to see if there’s something that I’m missing which… I don’t think there is.

*b*. *Fear of missing healthcare milestones and aging out*

While the idea of puberty blockers was positive for some families, some of the teenagers feared that their wait had been so long that they had already missed the best time to take puberty blockers. They were already dealing with a body that had changed. Yet, at the same time, they were not yet old enough for cross-sex hormones. As a result, they were stuck in a liminal space waiting for support they were both too late and too early for:

[Nell, parent]: Because of that [sighs] when I, um, [Xavier] can’t get any hormone treatments.

[Xavier, young person, 14]: Yeah! We basically missed the mark for blockers and we are still missing the mark for HRT.

[Nell]: Yeah.

[Xavier]: So there’s like… we’re that awkward middle position right now!

[Nell]: And [Xavier] can’t be given testosterone until… we’ve been seen in… Tavistock… So along with the dysphoria, this is what we’re really struggling with is certain parts of [Xavier]’s body are still growing.

[Xavier]: Yeah.

[Nell]: And things are expanding… And there’s nothing we can do about it.

One teenager, Oscar, spoke about the frustration of being on a long waiting list and he expected that there would be even more waiting once he was seen by the service. He was upset about the amount of time accessing support took, particularly as he had already gone through puberty while still waiting for his first appointment:

[If] I’m going to have blockers, you know it’s really not going to do anything, not a lot of point is it, now anyway? … I just hope it gets quicker whilst we’re there because we have to have (umm) you know more than one appointment to actually get on blockers. So I hope it just gets quicker and quicker right after that because it is actually ridiculous. Because they’re just adding months on and so on with you know it’s… what! Why? [sighs]

(Oscar, young person, 13)

Another teenager, Bodhi, who had been on the waiting list for almost two and half years believed his wait could have been even longer had he been referred to CAMHS first. While Bodhi experienced mental health difficulties, he felt that a prior referral to CAMHS was not relevant for him and would have delayed him being referred to GIDS. He described having to work to avoid a CAMHS referral:

Another thing that’s frustrating about it is most of the time they’ll try and… get you to go through the whole CAMHS process before you go through (umm) that Tavistock process. I managed to escape that because I convinced them to say that I didn’t have any like problems, like my mental health wasn’t impacted by me being trans. Um, which is still mainly the same today, like I have [laughs] it’s… it’s kind of sad to say [laughs] but I have bigger issues outside of being trans! [laughs]… I have worse things to worry about! [laughs].

(Bodhi, young person, 15)

Bodhi’s mother was also frustrated by the layers of waiting and spoke about never being able to plan for development and social milestones because they were still waiting for their first appointment:

I find it extraordinarily frustrating… I get angry really because I just feel… there’s obviously a massive growing need for this type of service in the NHS, but there seems to be no desire from them to expand those services… for these kids to have to be waiting for so long to get their referral it… I just think it’s cruel and it shouldn’t be happening. I find it very, very frustrating, um, the whole system… and just that not knowing when it will happen and you know you set these sort of milestones… what you want to happen by when… and there’s nothing you can do, you’re just completely out… it’s nothing that you can control.

(Melissa, parent)

Unsurprisingly, aging out of the service, when young people reached 17/18 years of age they could no longer access GIDS and were required to wait again on a waiting list for adult services, was a real fear among the older teenagers. The seeming endlessness of the wait for GIDS became even more pointless for these young people:

It was like ‘is the waiting list going to hurry up?’ … and it’s just got worse. Because even when you are seeing GIDS, it’s still like two year wait until any medical intervention at all and then by then I’ll about… like I’ll have out-aged that waiting list and I’ll be moved onto another. So there’s literally like no point.

(Roman, young person, 14)

I know people who have started going like… got on the GIDS waiting list when they were sixteen… got the referral like… got their first appointment by eighteen, been refused because they need to go in the adults and then waited another two years to get into the adults. And then more waiting and I feel really bad for all the people who are like that.

(Frankie, young person, 15)

*c*. *Can’t see a future while they’re stuck waiting*

With all the uncertainty and stuckness of waiting, some teenagers were unable to see themselves in the future beyond being able to further their transition. They could not imagine their future without feeling more comfortable within their bodies:

I don’t like to think about in the future what I’m going to do. Because it’s confusing. And I’m scared! I don’t want to be scared about my future.

(Griffin, young person, 13)

I just want to be me instead of this… like my mind-set is me, but I’m not in the right body and it’s… Uncomfortable and just unsettling not knowing whether I’m actually 100% going to get all that or not. And it’s the fact that I have to wait so long as well for all of it, it’s not nice.

(Frankie, young person, 15)

For some young people at times the distress of their situation became linked to self-harm and suicidality. When talking about her previous instances of self-harm, Iris stated: “Mum tells me it’s alright, but I know that it’s just going to keep happening unless I see the Tavistock” (Iris, young person, 11). Lucian (young person, 11) iterated that “waiting makes it worse”, because there was no certainty about when he could access support for his distress. Elena and Luna spoke about how they would call GIDS every day when they were first referred because of Luna’s suicidality until they realised they would not be given any support until their first appointment:

[Elena, parent]: At the beginning we were quite… you know we were calling the Tavistock every day, every day… Telling them that she was really distressed.

[Luna, young person, 14]: Yeah, I was like… suicidal.

[Elena]: Very… very suicidal and we were calling and they said oh we’re still seeing people from 2017. And I said what?! You know and… nothing.

Having supported Leith after he attempted suicide, Kendall (parent) wondered whether “there is a way of cascading that information and just… you know it’s not… a gender issue [it] isn’t a gender issue by itself… So if someone comes in with depression and self-harm and other issues, treat it like you would anything else”. In this way, young people like Luna and Leith might not be left without formal support when they were most vulnerable. Again though, Kendall understood there were systemic issues at play: “And I’m so aware that like the service is absolutely overwhelmed [laughs]”.

### 3.3. Suggestions for Support While Waiting

Finally, families’ narratives pointed to practical ways that GIDS could help them while on the waiting list. For some parents, even a brief conversation with someone from the service who told them they were doing the right thing was enough to alleviate concerns they might have had in how they were supporting their child: “I spoke to a man [at the service last… the beginning of the year I think it was… And he asked what we were doing, I said we were very much [Ronnie] led and he said perfect, everything we were doing was brilliant” (Ingrid, parent). This was especially important for parents given their exposure to the negative media portrayals about transgender and gender diverse children, and how gender binaries (where boys align with masculine tropes and girls with feminine ones) were presented. Gwen recalled wondering if she’d done the right thing in supporting her child’s transition when her child wasn’t interested in ‘girly’ clothes even though she identified as a girl. (Gwen, parent).

Carla would have liked to talk to someone from the service when her child was first referred to discuss how she could better support her child while they waited. She had found it very stressful to be left entirely alone to figure out how to support her child and was worried about making mistakes:

I would like to have in the beginning was to have somebody to actually sit down with me and like I’d go through the questions in my head… and then somebody just… just tell me, right so you know what research has shown is that yeah children do that until a certain age… Blah, blah, blah, blah. But not… none of it happened. It’s like… so it’s like in the end you would just left to our [own] device! [laughs]

(Carla, parent)

Another parent pointed out that an initial consultation when they were first referred to the service could have helped with the different practical and emotional issues they had navigated by themselves during the wait:

Possibly it would be good to have… when people are referred, like an initial consultation. Where they just give you a bit of advice and say you know yeah, its fine to call him she and… general kind of… advice like! [laughs] A kind of help book with kind of like where you could get underwear [laughs] from! Help centres. You know just… Just general kind of yeah you know we’ve found these sell great clothes, this website offers great advice, you know?

(April, parent)

Again though, how helpful parents found this sort of contact with service clinicians depended on the age of their child and whether they were distressed about puberty. Ingrid’s and Gwen’s children were two of the youngest interviewed, whereas Nova, as the parent of a teenager, had a very different experience. Nova spoke of her frustration on receiving a phone call from a clinician who did not help lessen her concerns about the impact of waiting on her child Oscar and could not speak directly Oscar:

We would like to be further along with GIDS. But honestly I… the most I read about them and the more challenges that they face, I just think it’s just going to be a… a futile activity. I… I’ve completely lost faith in the… in the whole process. For the fact that I had a clinician… can call me and speak to me for an hour, but can’t stop the process and have a Skype call with [GIDS clinician] for an hour!… You know mentally… these people are teenagers. Seriously? You’re missing the whole point people!

(Nova, parent)

Here, Nova wondered why it was deemed more important to talk to her than Oscar when the conversation did not mean they were going to be seen and thus taken off the waiting list. Nova’s concerns about the lingering impact of waiting on her Oscar’s mental health was mirrored by Marianne (parent): “I just think the mental torture and no matter… even by the point that they get seen you can never repair mental health like that”. For this reason, several parents felt that regular, even predictable, contact from the service to check in with the family would be beneficial:

At least they should sort of ring every two months or so and say how are you getting on whilst you’re waiting or even a letter to say we haven’t forgotten about you or anything… It is sad because you… your whole life is in… waiting for that one letter or that one phone call from them. And… oh! [sighs]

(Clara, parent)

Well just a phone call, just something, to say that you’re nearly there… So if yeah if we’re on the waiting list or… it’s got to be coming up two years… And we’re still waiting, so have they forgot about us? [laughs]?

(Goldie, parent)

With the type of check-in that that Clara and Goldie described, the family might still feel stuck on a waiting list but it might help to avoid them feeling abandoned or forgotten.

April also commented that with very little contact from the service prior to their first appointment, people had little idea where they were in the queue or what to expect at their first appointment. She suggested that the service give some even approximate indication of when they might receive their first appointment. Nell also spoke about the stress of feeling abandoned and forgotten and suggested that even receiving an email from the service every six months would provide some reassurance:

Just to let them know they’re not forgotten about… they’re still on someone’s radar… an email doesn’t cost much or you know maybe a couple of hours of someone’s time every six months. But it would really… it would really help some of the kids’ mental states”.

(Nell, parent)

Nell also wanted some indication of what she and Xavier could expect at their first appointment. She believed this would help them both feel more confident about the service and avoid the appointment feeling overwhelming or interrogatory:

But it starts to build that relationship and sort of the trust bonds… Instead of turning up on the first day, not knowing what’s going to happen because… you know even getting a guideline a month before of this is what to expect in the first meeting would give us time… for us specifically would give us time to talk about it… what questions you’re going to be asked, what to expect, instead of sitting there and feeling overwhelmed… By being bombarded with questions.

(Nell, parent)

Parents who knew their children worried about their GIDS appointment thought informal check-ins where their child could talk with a clinician would not only help maintain a sense of wellbeing during their wait, as well as manage their stress by knowing that the appointment could be like:

Just check in on them, how are things going with you, have you had your CAMHS appointment, is there anything that you think that me as a Tavistock healthcare professional… is there anything you want to ask me direct, anything I can help you with… give those kids hope that they’re not just being left to one side… a family meeting, you know so help some of those parents who are struggling to understand it… what their child’s going through… if the child was willing to have them in on it, obviously it’s the child’s choice. (umm) But yeah it might help them hearing things that they wouldn’t normally talk about with their parent, maybe to help them start to understand. So… yeah. I don’t know! [laughs]

(Celia, parent)

…you know to be able to have somebody to speak to and you know… maybe other ways of dealing with situations and things like that on [J]’s part. There’s things that you’re worried about isn’t that you probably want to speak to them about? But he has said that he’s going to be nervous to speak to them about personal things which is understandable.

(Francesca, parent)

Increased use of video calls during the lockdown also made parents realise this could be an effective way to have appointments where face-to-face involvement wasn’t required, “to help them get more appointments done quicker, so nobody’s travelling” (Celia, parent)

Other parents suggested being offered a helpline to call that was staffed by people who understood the issues they might be experiencing and could offer advice for how to cope with any difficulties while they were waiting:

But it could be even something as simple as… as a helpline… So no a helpline that… which give you… doesn’t need to give you… because obviously if you’re very lonely in the beginning and you feel very lost, if you’re like… there’s nobody there! [laughs] And yes! Basically there isn’t! [laughs]… So I think if there are some types of helpline. And you could just go through it and then say you know how long has he been like that for? Etcetera, your main questions, things like that. And then as long as it was like a psychologist, someone trained to do that. I know it’s not an easy thing to ask, but! [laughs]… So if you have somebody there, at least would give you some guidance. So I think it’s… it’s my main… my main concern and I think for future and for other families (umm) is that… that moment where your concerns… You know those moments where you get questions… Who do you turn to?”.

(Carla, parent)

In talking about their experiences of waiting, people demonstrated a sense of solidarity with other families also on the GIDS waiting list. Some wondered whether a community forum could be created for families on the waiting list so they could offer support to each other. This idea linked to the support some parents and young people had received from local LGBTIQ+ groups, or where some families had started their own group, but where there would be a link to GIDS information and more shared experiences. This was especially important to families who felt the GIDS website did not offer any useful information for people who were still on the waiting list:

I thought I’d just go on their website to see if there was anything that we could do. Maybe, I don’t know, I could drop in, have a phone call whatever. Nothing… their gatekeeping’s so heavy, just go and have a look at this information over there that anybody could look at. There was nothing for kids, parents, carers who were on the waiting list… Nothing!… my thought was there might be like a special club… Or even a group for people waiting. There wasn’t, so we sorted that out ourselves.

(Stella, parent)

And maybe even like some sort of GIDS community forum. Where the… there are actually patients of GIDS. So obviously you know if you’re… you’ve got like the unique password to log in, I don’t know but… But you’re all part of that, so you know that you are speaking to genuine people. And not just speaking to some man in his underpants! [laughs] Somewhere in the world! [laughs]

(April, parent)

When participating in other support groups, Larkin felt being able to talk to other families whose children had progressed further in their development and transition gave her more information to better plan for the future. This was the sort of support she felt should be available to anyone on the GIDS waiting list so families could ‘manage their expectations’ and know that there was a long road ahead of them:

And then one of the things I found beneficial from it is that I can see what other people who are further… who have an older child are going through and the challenges they’re having and why they’re having them. So it’s about insight into the likely future challenges. And so they don’t come as a surprise. Therefore, it’s actually managed my expectations earlier… Yeah, so it’s like knowing… knowing the shit that’s going to happen… Like if I don’t know, it’d be like god this is really frustrating and I’m really angry. And I can’t control… it’s out of my control and I’m frustrated and I’m angry for her. And it’s going to take months and weeks. I know all of that, so it’s… it’s just a case of helping her understand it and navigating… navigating her way through it. That’s really helpful… So you don’t know you know how long that black hole is before you see the light. Whereas actually OK well, I can see all these other people’s experiences, I’ve been monitoring and watching them for the last… ten years. So… what does that… you know I know that kind of direction and that path, so that visibility with that, that’s just what I’m like though in terms of being prepared… It just takes the stress away more than anything.

(Larkin, parent)

Having experienced how long the waiting list was, some parents of younger children also argued that young people who were most in need, such as those reaching puberty or who were struggling should be prioritised and that the waiting list should not always operate according to who had waited longest, but:

I don’t understand when there’s children that just come out and then they are self-harming, why they can’t jump the queue? Obviously we’re lucky that it’s come out early… So then we’ve got that time to maybe be seen before. But I know if [Lottie] come out later and she was self-harming I’ve not got the funds to go private… Like a lot of parents haven’t and I do think them children should get seen. Obviously we can wait a bit longer. I’d rather them get seen and wait a bit longer. Because they’re the ones that need the help. It is an emergency really because it’s life-threatening.

(Gwen, parent)

## 4. Discussion

This paper explored the experiences of children/young people and their parents while they continued to wait on the GIDS waiting list for support. For the young people about to start puberty, the lengthy waiting times were a significant cause of anxiety. For the young people who had already started puberty, the wait had arguably been too long for some of the medical support they were hoping to access. Feelings of frustration, hopelessness, and stuckness were common among the older adolescents and their families. Younger children may not always have been aware that they were waiting, but many of their parents worried about their future as the wait continued. These parents’ future thinking for their children was often bound in anxiety for whether they would be able to access support in time and what that would mean for the wellbeing of their child. Indeed, a strong narrative from parents of younger children was relief that they had managed to get on the waiting list so early because its length, and potential endlessness, was so well known among the community.

Indeed, waiting was painful in some way for almost all the families, whether it was just the parent who was worried about the future of their young child, or parent and an older adolescent worried about both the present and their future. Similar to the patient participants in Mulcahy et al.’s [6] study, many of the families wanted to be able to be more proactive in accessing treatment for their child or themselves, but were stymied in their attempts by being stuck on the waiting list. However, the participants waiting for treatment for cancer knew the promised treatment was definitely forthcoming. There was a fear among the participants in this study that the treatment and support they needed would not be accessible no matter how long they waited. This was not just due to the potential risk of aging out of GIDS and having to then join the waiting list for an adult service. This fear caused intense distress among the older adolescents, that they would be stuck for the rest of their lives in a body they were not comfortable or safe in; the imagery of ‘a ticking time bomb’ implies that it might well explode and lead to significant harm.

Families believed that the shift from ‘thinking time’ to ‘a ticking time bomb’ as the young person grew older, and the accompanying distress of this, could likely only be stopped when a young person started their appointments at GIDS. The majority of parents and young people understood why the GIDS waiting list was so long, and listed their knowledge around increasing numbers of referrals, the need for GIDS clinicians to have specialist knowledge, as well as the complexity of the work which led to staff turnover. However, an intellectual understanding of waiting can be very different to when you are living it. Parents felt helpless and hopeless as they watched their child suffer distress at their changing body, and young people felt distressed being stuck in such a body without knowing when and if they could be more in control of its changing.

Further, the endurance required by families for such a long wait, during such a physically and emotionally tumultuous time, highlights the privilege and luck that Ferrie and Wiseman [7] identify when a family realises they are able to access private healthcare. At the time of the first interview, no family reported accessing private healthcare, but the parents who believed they had the option to opt out of the NHS system for their child acknowledged their privileged position. It was seen as a last resort–they wanted to trust in the NHS–but the few parents with the funds were comforted by knowing they could access private care and this meant they could be hopeful for their child in ways that other parents could not.

To investigate whether waiting could be more of a positive experience, researchers in Australia trialled the First Assessment Single-Session Triage (FASST) clinic [25]. The FASST was found to be successful in its aims “to decrease wait time into the service, provide initial assessment and triage, and deliver information, education and support to patients who5are TGD and their families” [25] (p. 2). Young people who were able to access FASST showed an “increased sense of agency, contributed to by changes to outlook, validation, sense of self, and confidence” [25] (p. 7). In addition, Mulcahy and colleagues [6] highlighted the positive interactions many of their participants had with a cancer support group as a source of knowledge and comfort. In our study, parents of younger children particularly struggled with accessing information that they felt was reliable, practical, and from a trusted source. When parents had been able to access information like this from GIDS it appeared to be from their own proactive actions in telephoning and sending emails, very often on several occasions. Parents, and adolescents as they got older and understood more about GIDS, were beginning to question whether being ‘good’ or ‘patient’ made any difference as they were not sure when and if they would even be able to access treatment and support in time. This was especially exacerbated by the ‘ticking time bomb’ nature of puberty, and all the changes it entailed.

Fitzsimons and colleagues [2] demonstrated how their participants felt their lives were ‘on hold’ while they waited for coronary artery bypass surgery. Moving from the waiting list to a first appointment was simply the next step in accessing care. Parents and older adolescents knew they still had to undertake the GIDS clinical assessment process before being able to access any medical treatment. In line with the findings in Rickett et al. [13], the parents in our study, as well as some of the young people, also yearned for a greater sense of connection with other families in similar situations while in a holding pattern. In this way, the GIDS waiting list was a liminal space, “a state of in-between-ness and ambiguity” [26]. Living with hope in a liminal space means living “betwixt and between oscillating between the known and the unknown” [27]. Older adolescents especially demonstrated this liminal space-feelings of anxiety frustration, fear and stuckness, knowing there was a ‘ticking time bomb’ but not knowing when or if it would explode and what the aftermath might be.

### Limitations

The findings in this paper are based on the first wave of interviews in a longitudinal study with five waves of interviews in total. As such, it offers a ‘snapshot’ of families’ perceptions and experiences of waiting on the service waiting list. Future results will be published on analyses of the subsequent interviews to extend the findings presented here. We acknowledge that we do not have a large sample of families from minority ethnic backgrounds, but we did try to ensure our participant sample was as diverse as possible. While there are inevitably limitations to the generalisability of these findings, these narratives from parents, young people, and importantly young children demonstrate the continuing need for the voices of lived experience to be included in studies when examining experiences of healthcare systems.

## 5. Conclusions

Lee and colleagues (2020) argue that waiting is labour. It is “work demanded by an inequitable system, work that paradoxically further threatens the health and wellbeing of individuals seeking care… For those who are never able to receive services, or who receive them too late, they are given “false hope” in a system that is not able to provide enough resources within an adequate timeframe” [8] (p. 7).

The findings from this study highlight the variations in how waiting is experienced depending on the age of the child, and how distressed their body makes them feel. This study also demonstrates the further distress that can be caused when the waiting feels endless and there are no signs that families are still being considered within the service. Families often worried that they were no longer on the list at all or were lost in the system as they had not heard anything from the service in the months and years they waited. The specialist nature of the care these children and young people needed meant that this particular service was the only service these parents could access if they could not afford private care.

In this way, the support suggestions offered by the families, where check-ins could potentially provide reassurance that they were at least still on the list, were about finding ways to make the wait less distressing, not necessarily about making the wait shorter, which was more spoken about in terms of an ideal than a realistic option. Waiting for so long in a ‘liminal space’ also suggests that there needs to be better understanding of how to support families during their wait. This deserves more attention in order to, as far as possible, mitigate against the harmful impacts of long waits on children and their families.

## Figures and Tables

**Table 1:** Participant demographic data.

	Participants’ Pseudonyms		Young Person Age at Interview	Young Person Pronouns at Time of Interview	Minority Ethnic Identity	Autism Diagnosis	English Indices of Deprivation 2019
1	Parent	Dorothy					Mid
	Young person	Asher	12	He/him mostly but still occasionally used she/her	No	No	
2	Parent	Gia					Mid
	Young person	Matteo	8	He/him mostly but still occasionally used she/her	No	No	
3	Parent	Ruby					Low
	Young person	Everly	8	He/him	No	No	
4	Parent	Bonnie					Mid
	Young person	Alana	9	She/her	No	Yes	
5	Parent	Ingrid					Low
	Young person	Ronnie	6	He/him	No	Yes	
6	Parent	Gwen					Low
	Young person	Lottie	5	She/her	No	No	
7	Parent	Pandora					Low
	Young person	Lemon	6	She/her	Yes	No	
8	Parent	Brianna					High
	Young person	Frances	11	Parents still use she/her, young person likes he/him but doesn’t use all the time	No	No	
9	Parent	Clara					Mid
	Young person	Belle	7	She/her	No	Yes	
10	Parent	Bea					Mid
	Young person	Amara	12	She/her	No	Yes	
11	Parent	Fiona					High
	Young person	Poppy	9	She/her	No	Yes	
12	Parent	April					Low
	Young person	Peyton	7	Parents still use he/him more, but young person prefers she/her	No	Yes	
13	Parent	Hannah					Mid
	Young person	Daisy	7	She/her	No	No	
14	Parent	Carla					Mid
	Young person	Jonty	6	She/her	Yes	Yes	
15	Parent	Rowena					Mid
	Young person	Gaia	9	She/her	Yes	No	
16	Parent	Caroline					High
	Young person	Miles	14	He/him	No	No	
17	Parent	Nova					Mid
	Young person	Oscar	13	He/him	No	Yes	
18	Parent	Cleo					Low
	Young person	Ed	10	He/him	Yes	Yes	
19	Parent	Larkin					High
	Young person	Monroe	8	She/her	No	No	
20	Parent	Maryka					Low
	Young person	Riley	12	He/him	No	No	
21	Parent	Blythe					Mid
	Young person	Jordan	6	He/him but she/her still used a lot	No	No	
22	Parent	Ines					High
	Young person	Dylan	14	He/him	No	No	
23	Parent	Francesca					Mid
	Young person	Otto	10	He/him	No	No	
24	Parent	Felicity					Low
	Young person	Griffin	13	He/him	No	No	
25	Parent	Roxanne					Low
	Young person	Hallie	12	She/her	No	No	
26	Parent	Pippa					Low
	Young person	Charlie	7	He/him	No	No	
27	Parent	Rose					Mid
	Young person	Davie	12	He/him	No	No	
28	Parent	Marianne					Low
	Young person	Iris	11	She/her	No	No	
29	Parent	Melissa					Mid
	Young person	Bodhi	15	He/him	No	No	
30	Parent	Stella					Mid
	Young person	Frankie	15	He/him	No	No	
31	Parent	Aimee					Mid
	Young person	Nat	15	They/them	No	No	
32	Parent	Jessie					Mid
	Young person	Theo	9	He/him	No	Yes	
33	Parent	Elodie					High
	Young person	Felix	7	He/him	Yes	Yes	
34	Parent	Goldie					Mid
	Young person	Lucian	11	He/him	No	Yes	
35	Parent	Nell					Mid
	Young person	Xavier	14	He/him	No	No	
36	Parent	Celia					Mid
	Young person	Roman	14	He/him	Yes	No	
37	Parent	Kendall					Low
	Young person	Leith	14	He/him	Yes	No	
38	Parent	Elena					High
	Young person	Luna	14	She/her	Yes	No	
39	Parent	Dawn					High
	Young person	Rafferty	15	He/him	Yes	No	

## Data Availability

In order to maintain confidentiality and privacy, the data and datasets generated in LOGiC-Q will not be made available as participants in the study did not agree to this, and after advisement from the study PPI group.
